# Challenges and facilitators of transition from adolescent to adult HIV care among young adults living with HIV in Moshi, Tanzania

**DOI:** 10.1002/jia2.25406

**Published:** 2019-10-25

**Authors:** Rita V Masese, Julia V Ramos, Leonia Rugalabamu, Severa Luhanga, Aisa M Shayo, Kearsley A Stewart, Coleen K Cunningham, Dorothy E Dow

**Affiliations:** ^1^ Duke Global Health Institute Duke University Durham NC USA; ^2^ School of Medicine Johns Hopkins University Baltimore MD USA; ^3^ Kilimanjaro Christian Medical Centre Moshi Tanzania; ^4^ Division of Pediatric Infectious Disease Duke University Medical Center Durham NC USA

**Keywords:** Transition of care, young adults, youth, challenges, facilitators, HIV, Tanzania

## Abstract

**Introduction:**

Scale up of anti‐retroviral therapy has enabled millions of children infected with HIV to survive into adulthood, requiring transition of care to the adult HIV clinic. This transition period is often met with anxiety and reluctance. Youth who fail to transition may create strain on capacity in the pediatric and adolescent clinics or result in individuals dropping out of care entirely. This study examined challenges and facilitators to the transition among young adults living with HIV in Moshi, Tanzania.

**Methods:**

From April to June 2017, in‐depth interviews were conducted with young adults aged 18 to 27 years living with HIV in order to capture the spectrum of experiences from pre‐transitioning youth to those who successfully transitioned to adult care. Young adults were purposively recruited based on prior study enrollees and recommendations from healthcare staff. Recruitment occurred in the adolescent, adult HIV and the prevention of mother to child transition clinics at Kilimanjaro Christian Medical Centre. Two separate in‐depth interviews were conducted with eligible participants. Medical records were reviewed retrospectively to collect information on HIV‐related outcomes.

**Results:**

In‐depth interviews were held with 19 young adults. Participants mean age was 23.8 years (interquartile range 22.2 to 26.3 years); 53% were female. Most (78.9%) participants had been receiving anti‐retroviral therapy for nearly a decade and 72.2% were virologically suppressed (HIV RNA <200 copies/mL). Barriers to transition included fear of losing peer networks formed in the adolescent clinic, the abrupt manner in which young adults were asked to transition, stigma, financial constraints and a lower quality of care in the adult clinic. Facilitators of transition included family and social support, positive perspectives on living with HIV and maintenance of good health. Recommendations for transition included transition preparation, transition as a group and adoption of desirable aspects of the adolescent clinic (peer networks and education) in the adult clinic.

**Conclusions:**

Transition is a complex process influenced by many factors. As the number of young adults living with HIV continues to grow, it is vital to develop a transition protocol that addresses these challenges and is feasible to implement in low‐resource settings.

## 1 Introduction

In 2016, approximately two million adolescents about 10 to 19 years of age were living with HIV worldwide 1, 2. Eighty‐four per cent of these adolescents lived in sub‐Saharan Africa 1, 2. The East African nation of Tanzania is among the top five countries in the world for overall adolescent burden of HIV 3, 4, 5. Scale‐up of anti‐retroviral therapy (ART) has enabled an increasing number of young adults living with HIV (YLWH) to survive into adolescence and adulthood 1, 6. In addition to the physical, emotional and psychosocial challenges all adolescents face, YLWH face distinct challenges such as commencement or continuation of lifelong ART, drug side‐effects, retention in care, disclosure and mental health challenges associated with living with a stigmatizing chronic disease 7, 8, 9, 10. These challenges influence adolescents’ quality of life and social relationships making them more prone to high‐risk behavior and poor ART adherence, thereby increasing their susceptibility to disease complications and mortality 9, 11. Compared to children and adults, adolescents have worse ART adherence, lower rates of virologic suppression and higher rates of viral rebound after an initial period of suppression 1, 8, 12. For much of their childhood and adolescence, YLWH attend family‐centred and adolescent clinics where they not only receive medical care, but form close‐knit bonds with their care providers and peers. The supportive care system becomes synonymous to an external “family,” especially in the context where YLWH are orphaned 9. Adult services, by contrast, lack peer‐support services, making the transition to adult care a daunting task 13, 14, 15. Failure of transition results in an adolescent treatment bulge, further straining overburdened adolescent care facilities 6.

Current models for transition tend to focus on development of individualized transition protocols to assess readiness and identify barriers for each patient 13, 14, 16, 17. However, this is a resource‐intensive approach with limited feasibility in low‐resource settings 13, 14. Transition models from Africa have explored the use of peer counselors, social media interventions and financial incentives 9, 13. However, these models lack data on transition outcomes 9, 13.

The Health Care Transition Research Consortium (HCTRC) transition model was recently developed as a guiding framework for transition research and practice 18. The framework highlights the interaction between various factors at the individual, family/social, healthcare system and environmental domains that influence healthcare transition among youth with chronic conditions 18. This study aimed to describe the challenges and facilitators of transition from the perspective of the HCTRC framework. These findings will help inform a transition protocol that accommodates youths’ perspectives and may better facilitate retention of YLWH in the health system.

## 2 Methods

### 2.1 Setting

This study was conducted from April to June 2017 at the Kilimanjaro Christian Medical Centre (KCMC), the third largest hospital in Tanzania with a catchment area of approximately 15 million people. The hospital has an adolescent‐specific HIV clinic called Teen Club that targets youth 12 to 24 years of age and meets one Saturday each month. In addition to routine doctor visits and pharmacy refills, Teen Club activities include education sessions and social events. Approximately 250 youth attend Teen Club, most of whom acquired HIV perinatally 19. Current practice at KCMC encourages YLWH to transition to an adult clinic based on the following criteria: reaching age 25, becoming pregnant, marrying or exhibiting behavior considered to have a negative influence on peers such as substance use or having sex with fellow youth in Teen Club.

### 2.2 Participants

Young adults were eligible to participate in the study if they were: (a) 18 years or older, (b) currently attending Teen Club or the adult or prevention of mother to child transmission (PMTCT) HIV clinics after transitioning from Teen Club, (c) willing to speak about their experiences and d) able to provide informed consent. Recruitment was done purposively based on prior study enrollees 20, 21 and recommendations from the Teen Club healthcare staff. Eligible participants were approached, after clinic visits, by study team members (LR and SL) and informed about the study. The aim was to recruit 20 young adults into three groups: Group A (Transitioned): (i) four young adults who aged out of Teen Club and successfully transitioned to the adult clinic; (ii) four young adults who attempted to transition, but were unsuccessful and returned to Teen Club; Group B: six young adults who transitioned to the PMTCT clinic due to pregnancy; Group C: six youth who were soon to transition based on age (20 to 23 years old).

### 2.3 Data collection

Two female Tanzanian research assistants (SL, LR) trained in qualitative methods conducted in‐depth interviews (IDIs). The same interviewer conducted two separate IDI’s with the eligible participant in order to strengthen the internal reliability of the interview. The first interview was exploratory and aimed to establish rapport; the second interview was designed to probe responses from the first interview in order to obtain a deeper understanding of the participants’ responses. Both interviews from each participant were transcribed, translated to English and included in the present analysis.

IDIs were conducted in Swahili and ranged from 40 to 75 minutes in duration. The interval between the first and second IDI ranged from one to fourteen days. Research assistants utilized a semi‐structured interview guide with open‐ended questions and targeted follow‐up probes 22, 23. The interview guide was adapted from a prior study 11 and reviewed in a focus group with key stakeholders prior to interviewing participants. Questions focused on the participants’ perception of health, maturity, living with HIV, stigma, degree of medical autonomy, transitions in school and clinic care, perception of quality of care in Teen Club and the adult clinic and recommendations to improve transitions in care (Data S1: In‐depth interview guide). Participants received reimbursement for lunch (4000 TZ shillings/approximately 2 USD) and travel costs (2000 to 10,000 TZ shillings/approximately 1 to 4 USD) depending on the distance travelled to the interview site. Participants’ medical records were reviewed to obtain date of birth, year of HIV diagnosis, date of ART initiation, mode of HIV acquisition, date of transition, current ART regimen and most recent HIV RNA near the time of interview.

### 2.4 Data analysis

The IDIs were audio‐recorded, transcribed *verbatim* in Swahili and translated into English. NVivo version 11 software was used to organize, manage and code the data. The principal investigator (DED), interviewers (SL, LR) and two other study team members (RVM, JVR) read all the transcripts. Team members wrote summary memos and the transcripts were discussed in detail by the research team. Data saturation was anticipated after 20 interviews based on prior qualitative work in this population and transcripts were analysed in each group until no new insights were obtained 11, 22, 23. During the interview, some participants described experiences contrary to their pre‐defined group and were analysed according to their narrative. Recruitment ended at the point of data saturation.

We used a simplified grounded theory approach for data analysis 24. Codes were developed deductively (*a priori*) from topics in the interview guide and inductively (emergent findings) from the interview transcripts. Two researchers (RVM, JVR) independently coded portions of the interview transcripts, discussed and agreed upon coding decisions. Codes were then compiled into a codebook and applied to all transcripts. The coded transcripts were arranged into categories and subcategories based on similarities and differences among the study participants. The study adhered to the consolidated criteria for reporting qualitative research (COREQ) guidelines (Data S2: COREQ Checklist).

Quantitative data from the medical record abstractions were entered into Research Electronic Data Capture (REDCap). STATA version 15 software was utilized to conduct descriptive analyses. Viral suppression was defined as HIV RNA of <200 copies/mL. Perinatal HIV transmission was identified by self‐reported acquisition of HIV from the mother or confirmation of HIV diagnosis before 12 years of age with maternal death or positive maternal serostatus; otherwise status was categorized as unknown. Date of transition was deduced from retrospective chart review based on the day of clinic attendance (Teen Club is on Saturday; Adult on weekdays) or referral due to pregnancy.

Ethical approval was obtained from the Institutional Review Boards at KCMC, Duke University Medical Center and the Tanzania National Institute for Medical Research. All participants provided written, informed consent prior to data collection.

## 3 Results

### 3.1 Participant demographics

Nineteen participants were approached by a member of the research team, and all agreed to participate. All but one completed both interviews. These participants represented experiences from three stages of transition; however, some participants described experiences that differed from the category for which they were recruited. One male transitioned to PMTCT instead of the adult clinic to accompany his pregnant partner; three participants thought to be at pre‐transition had previously been asked to transition, but were unsuccessful. Participants’ ages ranged from 18.8 to 27.4 years (interquartile range 22.2 to 26.3 years). Fifteen participants were infected perinatally; four by unknown means. Slightly more than half of study participants (53%) were female. Nearly three‐quarters (72.2%) were virally suppressed (Table 1).

**Table 1:** Results of retrospective chart review

	Total (n=19)	Group A: transitioned to adult clinic (n=9)		Group B: transitioned to PMTCT clinic (n=7)	Group C: pre‐transition (n=3)	
		Succeeded (n=2)	Failed/refused (n=7)			
Age at the time of Study, mean (IQR)	23.8 (22.2 to 26.3)	27.1 (26.9 to 27.3)	23.4 (22.6 to 24.1)	24.6 (22.8 to 26.8)	20.9 (20.3 to 22.0)	
Gender, n (%)						
Female	10 (53)	2 (100)	1 (14)	6 (86)^a^		1 (33)
Age at HIV Diagnosis, mean (IQR)	11.9 (10.1 to 15.0)	10.8 (5.0 to 16.6)	11.1 (10.1 to 13.0)	13.2 (11.9 to 15.6)		11.6 (8.8 to 14.6)
Duration of ART in years Mean (IQR)	9.8 (9.2 to 11.9)	10.6 (9.3 to 12.0)	10.3 (10.0 to 11.8)	9.8 (8.7 to 12.2)		8.2 (5.8 to 10.9)
Age at time of transition, mean (IQR)		25.7^b^	21.5^b^	22.2 (20.6, 24.4)		N/A
ART Regimen, n (%)						
First line	11 (58)	1 (50)	6 (86)	3 (43)		1 (33)
Second line	8 (42)	1 (50)	1 (14)	4 (57)		2 (67)
Most recent HIV RNA<200 copies/mL, n (%)	13 (72.2)	2 (100.0)	5 (71.4)	5 (83.3)^c^		2 (66.7)

IQR, interquartile range.

a One male participant transitioned to the PMTCT with his pregnant partner

b Only one participant with deduced date of transition from file

c One participant had no documented HIV RNA prior to the study.

### 3.2 Interview themes

Analysis of transcripts from participants across the different stages of transition revealed similar and overlapping themes. The major themes identified were barriers and facilitators to transition (Table 2) and recommendations for transition of care (Table 3). Barriers and facilitators to transition were categorized into domains outlined by the HCTRC transition model in Table 2.

**Table 2:** Barriers and facilitators to transition and corresponding quotes

Theme^a^	Quotes
Barriers to transition	
Individual domain	
Financial constraints	“It’s hard for me. When we were told [to move], we were told that, for those who don’t have insurance, one has to pay five thousand in the adult clinic. Many people stopped taking medication. They said that if life is a matter of paying five thousand, they would rather stay home.” (22 years, male, failed transition)
Family social support domain	
Isolation/loss of peer network	“When I was required to move, meaning there are those who were my friends, who were seventeen [years old], maybe you are used to each other, you have stayed there maybe three or four years, and then you are required to move and leave one behind. So the friendship dies. So it could be that there are things that you thought about, you were helping each other, you can’t find them, because they come on Saturdays, you will be coming on Monday and that’s when there is a difference.”(22 years, female, failed transition) “Then I go to the adult CTC and meet grown ups I mean you won’t be comfortable as the way you stay with your fellow youths. You know grown ups are different from youths so I don’t know how it is going to be.” (20 years, female, transitioned to PMTCT)
Stigma	“Honestly when they were told to move to adult clinic most of them refused. They started saying from there that you will meet people who know you from the street who never knew you are infected and they start stigmatizing you. ‘Even a child of so and so,’[…] so from there is when most of the youth started refusing but they told them as per their age, ‘You will go there and receive care as you have been receiving from here.’ They accepted but after some time, the second month, the third, they met and later they got scattered, I don’t know where others went, I don’t know if they are here but they come on different days.” (27‐year‐old, female, successful transition)
Healthcare system domain	
Manner of transition/lack of preparation	“It was announced. It was announced to all youth. There was no one who did not know about it. It was announced publicly and not in secret or via fliers. We were called and told if you have reached a certain age you will move to the adult clinic. And we were given a reason, it’s not like we were not given a reason. We were given more details about the reason for transition after inquiring more about why we were being moved. We were told it’s because of this, this and this. ‘So you mean we are moving because of that?’ We agreed with the decision because we were grown up and those were children and we would have taught them things they weren’t ready for.” (22 years, male, failed transition)
Quality of care in the adult clinic	“The number of people there is one of the factors that cause the time taken to be long” (21‐year‐old, male, pre‐transition) “Nurses and doctors should be added. Other times you can find a few doctors and a few people getting the files. They need to increase the doctors and nurses” (27‐year‐old, female, successful transition)
Unfavourable adult clinic days	“It is not good, it is not good at all, I mean I am thinking it was not a good idea, they should not make the clinic day during the week because many people are not free. They are in school, at work, everywhere. Weekends should be good.” (24‐year‐old, male, failed transition)
Lack of education seminars and social events in the adult clinic	“The adult CTC [adult clinic] is different somehow, I mean things that are done in the CTC are different. In the youth clinic there are things that we used to be taught, that used to help, but after transition to the adult clinic you just come give out your card and go to doctor, take your medicines and go home. So there is nothing about seminars, or what. There is none.” (26 year‐old‐female, transitioned to PMTCT)
Facilitators of transition	
Individual domain	
Maintenance of good physical, mental and emotional wellbeing.	“Another responsibility to my health is making sure that my health is stable because if it’s unstable I will take several steps back in life. I will not be able to go to work when I am ill, I wouldn’t be able to do anything when I am ill. I will go back on my progress and even economically.” (27‐year‐old, female, successful transition)
Positive perspective of HIV and self‐acceptance	“You see here I am HIV positive, this is not the end. I am not dying. There are accidents, there are those who get cancer, they die but when they adhere to medicine well, they get treated very well, and they live well, as long as you eat food and you check your CD4 on time.” (27‐year‐old female, transitioned to PMTCT) “I have accepted myself and I have not lost hope. Had I lost hope every time they speak that way, you may find that I would have already stopped taking my medicines and had died. But despite all these things that I face, it pains only at the time when they talk about me, but once I am out of there I forget about it.” (26 year old, female, transitioned to PMTCT)
Family social support domain	
Family social support	“My parents are important especially my father is important because he is the one who looks after me and takes care of me even when I am sick .He is the one who supports me by paying the hospital charges, transport charges, food and other things and that’s why he is important. If it weren’t for him, maybe I wouldn’t be here talking to you right now.”(26 year old, female, transitioned to PMTCT)

a *A priori* findings included financial constraints and loss of peer network as barriers to transition. Other themes were emergent findings.

**Table 3:** Recommendations for transition

Recommendation	Details	Representative quotes
Recommended age for transition	25 to 26 years with allowance of extension if youth feel ill‐prepared.	“Per my thoughts they should transition at 25 years. It is the age when youth are now grows‐ups and understand themselves.” (22 year old, female, pre‐transition)
Transition Activities	1. At least one year of transition preparation. 2. Transition youth as a group. 3. Youth mentorship in the adult clinic. 4. Permission for transitioned youth to visit friends in Teen Club.	“They need to try to schedule our dates such that three or four people can come on the same day. You can go and find that you are alone with people you are not used to.” (27‐year‐old female, successful transition)
Adoption of certain aspects of Teen Club to the adult clinic	1. Educational seminars on topics such as HIV, ART, sexual and reproductive health. 2. Psychosocial support groups. 3. Group activities such as soccer matches, hikes, etc. 4. Increase the number of adult clinic physicians. 5. Division of the adult clinic into age categories.	“They should include sports. When we go there we should have our sports and we should get taught. We should cooperate when we are together as groups.” (20‐year‐old, female, transitioned to PMTCT)
Transition to PMTCT clinic	1. Counselling and provision of family planning. 2. Creation of support groups in the PMTCT clinic. 3. Improvement in continuity of care with explanation of reasons for ART changes.	“You get the tablets you need, you get the tablets you use every day. They don’t get changed [at the PMTCT clinic]. And if changing is necessary, they get changed and you are told. They don’t do it as a secret, they tell you.” (20‐year‐old, female, transitioned to PMTCT)

### 3.3 Barriers to transition

Most participants were distressed by the abruptness in which they were asked to transition. The process generally consisted of the clinical staff assembling young adults into a room during a regular Teen Club day and announcing they were to transition to the adult clinic the following month. Pregnant females were asked to transition upon realization they were pregnant. Beyond the announcement, most participants felt that there was little effort to prepare them for the transition. This lack of preparation created anxiety within a majority of the young adults.

Stigma was another prominent barrier to transition. Some participants feared that adult HIV patients would recognize them and disclose their status to the community. They also feared that adults would assume they were infected through high‐risk behavior, and this deterred them from moving to the adult clinic.

Financial constraint was also reported as a transition barrier for a majority of the participants. Despite the provision of free ART, patients had to pay 5000 TSH (approximately 2 USD) for an adult HIV clinic visit, whereas Teen Club was free. Most participants viewed the adult HIV clinic fee as unaffordable; however, most participants were willing to pay 1000 to 2500 TSH (approximately 0.5 to 1 USD) to continue care at KCMC rather than transition to an alternative government clinic with no fee.

Some participants stated differences in the quality of care between the adult clinic and Teen Club as another reason they resisted transition. Participants who transitioned reported high patient volumes and few physicians, which led to long wait times in the adult clinic. Participants also complained about communication barriers they experienced with adult clinic providers. Many reported that physicians were “very tired,” “didn’t listen to problems in detail” and were “in a hurry;” and they felt nurses lacked empathy and compassion.

Differences in clinic days and activities were also identified as barriers to transition. Adult clinic was held on weekdays, while Teen Club was one Saturday every month. Weekday clinics were challenging for some participants who were in school or employed. Additionally, Teen Club held activities such as educational seminars where youth learned about nutrition, ART adherence and other health maintenance strategies. The educational seminars provided a platform for bonding and formation of peer support networks, and many felt discouraged by the possibility of losing their friends from Teen Club. Similar seminars did not occur in the adult clinic. Most participants were concerned about being isolated with “no one to talk to” as they waited to receive care in the adult clinic.

The transition of pregnant participants to PMTCT highlights the quality of care barrier. Female youth who became pregnant were transitioned to the PMTCT clinic regardless of age. Participants were hesitant to transition due to unfamiliarity with and anticipated stigma in the PMTCT clinic. Despite feeling hesitant initially, all participants reported receiving good care in the PMTCT clinic. One participant remarked that the nurses were “compassionate and polite.” The quality, however, was not perfect. One participant expressed concern about the lack of continuity of care among Teen Club and PMTCT providers. In one instance, this gap in care led to changing ART with no reported indication for the regimen change:

*I don’t know why they change medicines and that is what confuses me. One day I was given yellow tablets. Another day I was given white tablets. They made me very itchy. I went to the pharmacy and I was told that I have to see the doctor and I wasn’t at Teen Club, I was at the PMTCT [clinic] and the doctor there didn’t know about all this so I had a very hard time.* (20 year old, female, transitioned to PMTCT)

After childbirth, young parents attended PMTCT for two years and were then asked to move to the adult clinic. However, the majority of these new parents wanted to be transferred back to Teen Club instead of the adult clinic due to fears of isolation and stigma as detailed above.

### 3.4 Facilitators of transition

Participants who successfully transitioned mentioned receiving psychosocial and financial support from family. One participant, referring to his family, stated, “Well, when it comes to reminding me to take medication, they remind me. Initially I used to be forgetful but they remind me to eat well. They remind me to eat so they have contributed in a big way.” (27‐year‐old, female, successful transition)

Participants who transitioned successfully demonstrated a positive perspective and an understanding and acceptance of their disease. Participants who had a positive outlook on living with HIV also demonstrated high self‐esteem, confidence and lack of internal stigma.

Most participants mentioned good health as a priority in their lives. Health was defined as being physically, mentally and spiritually well. Participants recognized the value of a balanced diet, physical exercise, stress relieving mechanisms and good ART adherence as components of sustaining good health. Participants who employed stress relieving mechanisms had adaptive emotional functioning and were more proactive about dealing with mental and emotional stress.

*Friends are important because we exchange ideas. Maybe I have thoughts and maybe when I talk about my issue, we advise each other. I wouldn’t be like this without friends. (27 year old, female, successful transition)*

*I don’t isolate myself. I don’t like isolating myself because when I am by myself sometimes, I think about many things that are hurtful. I cry and stuff but I associate with people. We go exercise together, we play, we do things. That helps me. (18 year old, male, pre‐transition)*

### 3.5 Recommendations for transition

Table 3 summarizes recommendations for transition. Participants suggested 25 to 26 years as the ideal age for transition with flexibility to transition later if they felt ill prepared. Participants also recommended structured transition activities, such as preparing for up to a year and moving as a group in order to maintain their support for each other. They also suggested adapting some of the group aspects of Teen Club, such as the education seminars and support groups, to the adult and PMTCT clinics.

## 4 Discussion

Transition of care among YLWH is a complex process influenced by many factors. The HCTRC framework provides a valuable guide to discuss the factors participants reported.

### 4.1 Individual domain

Individual factors that influenced transition included the participants’ recommended age for transition, perception of HIV and financial constraints. Several studies exploring the preferred age to prepare for transition had varied results 25, 26, 27. Initial transition preparation ranged from time of diagnosis to late teen years. The age for transition (25 years) proposed by participants in this study is older than the late teen or early twenties reported in most studies 25, 26, 28. Other studies proposed initiation of transition according to the youth’s level of maturity and self‐management capability 16, 29, 30, 31. Successfully transitioned participants in our study were older and perhaps exhibited more self‐efficacy at the time of transition. Those who failed transition may have been asked to transition due to behaviour, or were not prepared to successfully make the transition. Awareness and early preparation for transition is essential for successful transfer to adult care.

YLWH experience psychosocial stress from HIV‐ and non‐HIV‐related factors 32, 33. Studies note that YLWH have higher rates of anxiety and depression when compared to non‐HIV‐infected counterparts 32, 34, 35. Mental health interventions that equip YLWH with coping and stress‐relieving mechanisms may be effective in promoting a positive perspective on living with HIV, which is linked to good ART adherence and improved self‐care 6, 14, 20, 32. This interplay of mental health issues and health maintenance suggests a critical role for strategies that improve resilience for YLWH in facilitating transition of care 20, 32, 36, 37, 38.

Financial constraints are an important barrier of transition in both high and low‐resource settings 16, 31, 35. A systematic review by Tepper *et al* on transition outcomes in North America and Europe revealed that parental insurance coverage terminated around the time of transition, resulting in gaps in treatment before youth obtained medical coverage 35. In low‐resource settings, health insurance may be less common, but similar research findings show that direct financial support from parents and others dwindle as youth reach young adulthood, thereby increasing barriers to transition 13, 26, 33. Most participants in this study were still financially dependent on their families and could not afford clinic fees, which deterred them from transitioning to adult care.

### 4.2 Family social support domain

The family social factors identified in our study were stigma and family support. Stigma negatively affected seeking care, and was associated with poor ART adherence and reluctance to transition. Our results correspond with similar studies about transitioning YLWH 26, 39. Interventions tailored to increasing community awareness and addressing misconceptions about HIV may help mitigate stigma and facilitate social support to youth as they transition into adulthood 7. Additionally, this study highlights the importance of family support through financial assistance and psychosocial support, similar to other studies 32, 36.

YLWH also relied on peers from Teen Club, which promoted good healthcare maintenance, and feared that transition would bring loss of their peer support. The participants’ recommendations for group transition, peer mentors in the adult clinic and permission to visit friends in Teen Club would likely mitigate the sense of peer loss and ensure continuity of peer support in the adult clinic, thereby facilitating successful transition.

### 4.3 Healthcare system domain

The main healthcare system barrier, like other studies in low‐resource settings, was the overwhelmed adult clinic with long waiting times and few physicians 13, 15, 40. Healthcare facilities lack adequate resources to address structural barriers to transition. Teen Club and other adolescent care facilities are places YLWH do not want to leave 16, 41. Healthcare providers also lack training on transition which extends to lack of youth preparation for transition despite evidence suggesting that preparation improves transition outcomes 13, 14. Several studies in high‐resourced settings utilized transition readiness tools such as the Transition Readiness Assessment Questionnaire (TRAQ) 42 and the Social‐Ecological Model of Adolescent and Young Adult Readiness to Transition (SMART) 43 that measured youth's expectations, motivation, developmental maturity and other domains to determine if youth were ready for transition 30, 40. Unfortunately, most of these transition preparation tools are not applicable in low‐resource settings since they do not account for differences in social cultural norms, institutional norms and system level factors such as poverty and lack of adequate infrastructure and healthcare training. Further research is required to develop appropriate transition readiness tools that would be culturally relevant and applicable in low‐resource settings 13, 44.

Based on the KCMC hospital transition criteria, some YLWH who attend Teen Club were asked to transition to the adult HIV clinic if they reached a designated age or were thought to engage in behaviour considered to have a negative influence on peers. These criteria reflect a systems barrier that may discourage YLWH from reporting certain behaviours for fear of premature transition. Transition is a necessary process in order to accommodate the next cohort of children entering adolescence; however, without significant preparation, an evidence‐based transition protocol tailored to this setting and an environment that feels welcoming, YLWH are at increased risk of loss to follow‐up and disengagement with care altogether.

### 4.4 Environmental domain

Adolescents represent a neglected subgroup in Tanzanian health policy and government expenditure despite adolescents accounting for almost a quarter of the population 5, 45. There are no national guidelines that govern the transition of adolescents to adult care. This deficit reflects a fragmented healthcare system similar to most countries in sub‐Saharan Africa 45. Failure to develop and implement transition protocols at a national or even a hospital‐level results in arbitrary transfers to adult clinics with limited feedback about successes and failures 41.

Poverty and lack of education are important considerations in the environmental domain. The public education system often excludes youth from low socio‐economic status who are unable to pay school fees. Low levels of education translate to an inability to obtain stable income‐generating activities, perpetuating a vicious cycle of poverty that not only serves as a barrier to transition, but limits resources for transport to care, clinic fees and impedes overall healthcare maintenance 46.

## 5 Limitations

While this study provides valuable insight on the transition process, there are several important limitations. First, all participants in the study were recruited from Teen Club, the PMTCT and the adult HIV clinics at KCMC. Their experiences may be less generalizable to YLWH in other African settings who do not have access to specialty paediatric, adolescent or antenatal care. Second, KCMC may be unique in charging a fee for adult HIV care compared to other government facilities where many YLWH attend clinic. Financial strains, however, remain generalizable as transport fees and opportunity costs with time away from work or school are experienced widely. Finally, the study included participants who were infected perinatally as well as those whose mode of infection was unknown. It is possible that those infected perinatally may have differing opinions to those infected through high‐risk behaviour, though for this study sample themes were consistent across transition groups. Despite these limitations, this study was the first of its kind in Tanzania to investigate factors influencing the transition of adolescents to adult HIV care from the perspective of YLWH.

## 6 Conclusions

There are many barriers to transitioning from adolescent to adult HIV care, and they exist at multiple levels. There is a clear need to develop and test feasible transition protocols that take into account the perspective of YLWH and are designed for the African context. These transition protocols should address factors at each of the individual, family, social, healthcare system and environmental levels. Findings from this study will inform a transition protocol that better accommodates youth and other stakeholders’ recommendations to improve the transition process.

## Competing interests

All authors have no competing interests to declare.

## Authors’ contributions

D.E.D. designed the study and was in charge of direction, planning and fund sourcing J.V.R., D.E.D., L.R., S.L. deigned the data collection instruments, R.V.M., J.V.R., L.R., S.L., A.M.S., D.E.D collected and analysed data, D.E.D., J.V.R., R.V.M. wrote the manuscript with input from all authors, K.A.S., C.K.C. provided technical expertise in the methodology and edited the manuscript.

## Supporting information


**Data S1. **In‐depth interview guide.

**Data S2. **Consolidated criteria for reporting qualitative research (COREQ) guidelines.

## Data Availability

The dataset is openly available to researchers who contact the Duke IRB, the study’s principal investigator and meet confidentiality requirements (documentation of ethics training in conduct of human‐subject research). Dataset requests may be sent to Dorothy Dow (dorothy.dow@duke.edu)
