# Supplementary material for: Challenges and facilitators of transition from adolescent to adult HIV care among young adults living with HIV in Moshi, Tanzania
Source: J Int AIDS Soc. 2019 Oct 25;22(10):e25406. doi: 10.1002/jia2.25406 (PMC6813636; doi:10.1002/jia2.25406)
Supplement: Supplementary file 2 — Data S2. Consolidated criteria for reporting qualitative research (COREQ) guidelines. [file JIA2-22-e25406-s002.docx]

| **GROUP A ENGLISH (HAVE TRANSITIONED), PART 1** |
| --- |
| **IN DEPTH INTERVIEW PART 1** |
| Thank you so much for joining us today for your first of two interviews. During today’s interview and your interview in the future, we hope to talk about health care transition with you. Health care transition is a term used to describe when adolescent patients like you transfer care from Teen Club to the Adult CTC. Our conversation today will last between 45 minutes to an hour. In order to learn about different aspects of the transition process, we will discuss topics such as transition, social support, socioeconomic status, relationships, stigma, autonomy, ART adherence, and responsibilities. What you share with us today will be used to create a transition protocol, which will support adolescents throughout their process of transition. |
| I have a recorder here and I have turned it on. You don’t need to pay any attention to it, it will stay here during the interview. The recorder will help me capture all that you share with me today. What we discuss will remain confidential and only the study team will listen to your recording. We ask that you try not to use names during the interview in order to protect your privacy and the privacy of others. Before we begin, is it okay with you if I record this interview? |
| **ESTABLISHING RAPPORT/EXPLORING SELF PERCEPTIONS** |
| 1. Tell me a little about yourself. |
| 2. Tell me about school. |
| 3. Tell me about your income generating activity or goals for generating income if you are not already doing so. |
| **LIVING SITUATION AND SOCIOECONOMIC STATUS** |
| 4. Now I’d like to hear about your living situation. |
| **TEEN CLUB** |
| 5. Now I would like to talk about Teen Club. |
|  |
| **TRANSITION** |
| 6. Health care transition is a term used to describe when adolescent patients like you transfer care from Teen Club to the Adult CTC. Tell me about your transition process. |
| PROBES: |
| **ADULT CLINIC** |
| 7. What would your ideal Adult CTC look like? |
| **HEALTH** |
| 8. Now, I would like to learn about your health. |
| **SOCIAL** |
| 9. Now I would like to learn about the important people in your life. |
| **GROWING UP** |
| 10. Now I would like to know more about becoming an adult. |
| **CLOSING OF INTERVIEW** |
| 11. What else haven’t I asked you that you think I should know? |
| I would like to thank you for sharing your experiences with me today. Is there anything else before we conclude the interview? |
| **GROUP A ENGLISH (HAVE TRANSITIONED), PART 2** |
| **IN DEPTH INTERVIEW PART 2** |
| 1. Last time, we talked about what happened during your transition, who was involved, and what made you excited or scared. Today we would like to learn about your opinion regarding your transition process. |
| **ADULT CLINIC** |
| 2. Remember the ideal Adult CTC that you described to us during our interview last time? Today, we would like to learn how the real Adult CTC compares to your ideal clinic. |
| 3. What do you think about the current Adult CTC? |
| 4. What may stop you from going to the Adult CTC or taking your medicine? |
| 5. How was the process of transitioning to the Adult CTC? |
| **GROWING UP** |
| Remember when we talked about becoming an adult last time? Now I would like to learn a bit more about what being an adult means to you. |
| 6. Let’s talk about responsibility. |
| 7. Now I would like to talk about autonomy. |
| **PARENTING** (If no children, skip PARENTING section, reference Question 4 from Part I) |
| 8. Can you share with us how your HIV health care routine changed when you had a child? |
| **HEALTHCARE MAINTENANCE** |
| 9. Lastly, I would like to learn about your HIV health care. |
| **CLOSING OF INTERVIEW** |
| 10. What else haven’t I asked you that you think I should know? |
| I would like to thank you for sharing your experiences with me today. Is there anything else before we conclude the interview? |
|  |
| **GROUP B ENGLISH (TRANSITIONED DUE TO PREGNANCY), PART 1** |
| **IN DEPTH INTERVIEW PART 1** |
| **ESTABLISHING RAPPORT/EXPLORING SELF PERCEPTIONS** |
| 1. Tell me a little about yourself. |
| 2. Tell me about school. |
| 3. Tell me about your income generating activity or goals for generating income if you are not already doing so. |
| **LIVING SITUATION AND SOCIOECONOMIC STATUS** |
| 4. Now I’d like to hear about your living situation. |
| **TEEN CLUB** |
| 5. Now I would like to talk about Teen Club. |
| **PMTCT TRANSITION** |
| 6. Health care transition is a term used to describe when adolescent patients like you transfer care from Teen Club to an Adult clinic. Tell me about your transition process from Teen Club to PMTCT. |
| **TRANSITION TO ADULT CTC FROM PMTCT** |
| 7. What do you think about transitioning from PMTCT to Adult CTC? |
| PROBES: |
| **ADULT CLINIC FOR THOSE WHO HAVE NOT TRANSITIONED TO ADULT CTC** |
| 8. What would your ideal Adult CTC look like? |
| PROBES: |
| **HEALTH** |
| 9. Now, I would like to learn about your health. |
| **SOCIAL** |
| 10. Now I would like to learn about the important people in your life. |
| **GROWING UP** |
| 11. Now I would like to know more about becoming an adult. |
| **CLOSING OF INTERVIEW** |
| 12. What else haven’t I asked you that you think I should know?I would like to thank you for sharing your experiences with me today. Is there anything else before we conclude the interview? |
| **GROUP B ENGLISH (HAVE TRANSITIONED DUE TO PREGNANCY), PART 2** |
| **IN DEPTH INTERVIEW PART** |
| **PMTCT TRANSITION** |
| 1. Last time, we talked about what happened during your transition to PMTCT, who was involved, and what made you excited or scared. Today we would like to learn about your opinion regarding your transition process. |
| **TRANSITION** |
| 2. Last time, we also talked about transitioning to the Adult Clinic, what you have learned about it so far, and what makes you excited or scared. Today we would like to learn about your opinion regarding this transition process to adult clinic. |
| **ADULT CLINIC** |
| 3. Remember the ideal Adult CTC that you described to us during our interview last time? Today, we would like to learn how the real Adult CTC compares to your ideal clinic. |
| **GROWING UP** |
| Remember when we talked about becoming an adult last time? Now I would like to learn a bit more about what being an adult means to you. |
| 4. Now I would like to talk about responsibility. |
| 5. Now I would like to talk about autonomy. |
| **PREGNANCY** |
| 6. Can you share with us how your HIV health care routine changed when you got pregnant? |
| PROBES: |
| **HEALTHCARE MAINTANENCE** |
| 7. Now I would like to learn about your HIV health care. |
| **CLOSING OF INTERVIEW** |
| 8. What else haven’t I asked you that you think I should know? |
| I would like to thank you for sharing your experiences with me today. Is there anything else before we conclude the interview?   \| **GROUP C ENGLISH (HAVE NOT YET TRANSITIONED), PART 1** \| \| --- \| \| **IN DEPTH INTERVIEW PART 1** \| \| **ESTABLISHING RAPPORT/EXPLORING SELF PERCEPTIONS** \| \| 1. Tell me a little about yourself. \| \| 2. Tell me about school. \| \| 3. Tell me about your income generating activity or goals for generating income if you are not already doing so. \| \| **LIVING SITUATION AND SOCIOECONOMIC STATUS** \| \| 4. Now I’d like to hear about your living situation. \| \| **TEEN CLUB** \| \| 5. Now I would like to talk about Teen Club. \| \| **TRANSITION FOR THOSE WHO HAVE NOT TRANSITIONED** \| \| 6. Health care transition is a term used to describe when adolescent patients like you transfer care from Teen Club to the Adult CTC. What do you think about transition? \| \| **ADULT CLINIC FOR THOSE WHO HAVE NOT TRANSITIONED TO ADULT CTC** \| \| 7. What would your ideal Adult CTC look like? \| \| **HEALTH** \| \| 8. Now, I would like to learn about your health. \| \| **SOCIAL** \| \| 9. Now I would like to learn about the important people in your life. \| \| **GROWING UP** \| \| 10. Now I would like to know more about becoming an adult. \| \| **CLOSING OF INTERVIEW** \| \| 11. What else haven’t I asked you that you think I should know? \| \| I would like to thank you for sharing your experiences with me today. Is there anything else before we conclude the interview? \| \| **GROUP C ENGLISH (HAVE NOT YET TRANSITIONED), PART 2** \| \| **IN DEPTH INTERVIEW PART 2** \| \| **TRANSITION** \| \| 1. Last time, we talked about transition, what you have learned so far, and what makes you excited or scared. Today we would like to learn about your opinion regarding the transition process. \| \| **ADULT CLINIC** \| \| 2. Remember the ideal Adult CTC that you described to us during our interview last time? Today, we would like to learn how the real Adult CTC compares to your ideal clinic. \| \| **GROWING UP** \| \| Remember when we talked about becoming an adult last time? Now I would like to learn a bit more about what being an adult means to you. \| \| 3. Let’s talk about responsibility. \| \| 4. Now I would like to talk about autonomy. \| \| **PARENTING (if no children, skip this section).** \| \| 5. Can you share with us how your HIV health care routine changed when you had a child? \| \| **HEALTHCARE MAINTENANCE** \| \| 6. Lastly, I would like to learn about your HIV health care. \| \| **CLOSING OF INTERVIEW** \| \| 7. What else haven’t I asked you that you think I should know? \| \| I would like to thank you for sharing your experiences with me today. Is there anything else before we conclude the interview? \| |

**SWAHILI TRANSLATIONS OF INTERVIEWS**

| **KUNDI A SWAHILI, SEHEMU YA KWANZA** |
| --- |
| **SEHEMU YA KWANZA** |
| Asante sana kwa kujiunga na sisi leo kwa ajili ya mahojiano yako ya kwanza kati ya mawili. Wakati wa mahojiano ya leo na mahojiano yako wakati ujao, tunatumaini kuongelea mpito au mabadiliko ya matunzo ya afya na wewe. Mabadiliko ya matunzo ya afya ni maneno yanayotumika kuelezea wakati wagonjwa vijana wadogo kama wewe wanapohamisha matunzo toka ***Teen Club*** kwenda ***CTC*** ya Watu wazima. Mazungumzo yetu leo yatadumu kati ya dakika 45 hadi saa moja. Ili kujifunza kuhusu vipengele tofauti vya mchakato wa mabadiliko, tutajadili mada kama mabadiliko, msaada wa kijamii, hali ya uchumi-jamii, mahusiano, unyanyapaa, kujitawala, kufuata utaratibu wa ART au dawa za kupunguza makali ya VVU kwa uaminifu, na wajibu. Unachotushirikisha leo kitatumiwa kutengeneza itifaki ya uhamaji, ambayo itawasaidia vijana kwa kipindi chote cha mabadiliko. |
|  |
| **KUJENGA MAELEWANO/ KUPELELEZA UTAMBUZI BINAFSI** |
| 1. Niambie kidogo kuhusu wewe mwenyewe. |
| 2. Niambie kuhusu shule. |
| 3. Niambie kuhusu shughuli yako ya kuingiza kipato au malengo yako ya kuingiza kipato kama bado hujaanza kufanya hivyo. |
|  |
| **HALI YA MAISHA NA HALI YA KIUCHUMI KIJAMII** |
| 4. Sasa, ningependa kusikia kuhusu hali yako ya maisha. |
| **KLABU YA VIJANA WADOGO** |
| 5. Sasa ningependa kuongelea Klabu ya vijana wadogo. |
| - Niambie kuhusu Klabu ya Vijana. |
| **MABADILIKO/MPITO** |
| 6. Mpito wa matunzo ya afya ni maneno yanayotumiwa kuelezea pale wagonjwa vijana wadogo kama wewe wanahama matunzo/huduma kutoka Klabu ya Vijana kwenda CTC ya Watu wazima. Hebu niambie kuhusu mchakato wako wa mpito. |
| **KLINIKI YA WATU WAZIMA** |
| 7. Ungependelea CTC yako iweje? |
| 8. Sasa, nitataka kujua kuhusu afya yako. |
| **KIJAMII** |
| 9. Sasa ningetaka kujua kuhusu watu muhimu katika maisha yako. |
| **KUKUA** |
| 10. Sasa ningetaka kujua zaidi kuhusu kuwa kwako mtu mzima |
| **KUFUNGA MAHOJIANO** |
| 11. Ni kitu gani kingine ambacho sijakuuliza unachofikiri ninahitaji kukijua? |
| Ninapenda kukushukuru kwa kunishirikisha uzoefu wako leo. Kuna kitu kingine chochote kabla hatujafunga haya mahojiano? |
|  |
| **KUNDI A SWAHILI, SEHEMU YA PILI** |
| **SEHEMU YA PILI** |
| **MABADILIKO/MPITO** |
| 1. Mara ya mwisho, tuliongelea kile kilichotokea wakati wa kuhama kwako, nani alihusika, na nini kilikusisimua au kukuogopesha. Leo tungependa kujua kuhusu maoni yako kwa mchakato wa kuhama kwako. |
|  |
| **KLINIKI YA WATU WAZIMA** |
| 2. Unakumbuka ile CTC bora kabisa ya Watu wazima ambayo uliielezea kwetu kwenye yale mahojiano mara ya mwisho? Leo, tungependa kujua jinsi hiyo CTC halisi ya Watu wazima inavyofanana na ile kliniki yako ya mfano. |
| 3. Unafikiri nini kuhusu Kliniki ya sasa ya CTC ya Watu wazima? |
|  |
| 4. Nini kinaweza kukuzuia kwenda CTC ya Watu wazima au kumeza dawa zako? |
| 5. Mchakato wa kuhamia CTC ya Watu wazima ulikuwaje? |
| **KUKUA** |
| Unakumbuka wakati tulipoongea mara ya mwisho kuhusu kuwa mtu mzima? Sasa ningependa kujua zaidi kidogo kuhusu kwamba kuwa mtu mzima kuna maana gani kwako. |
| 6. Hebu tuongee kuhusu wajibu. |
| 7. Sasa ningependa kuongelea kujitawala / uhuru binafsi. |
| **MALEZI** (kama hana watoto, ruka kipengele hiki cha MALEZI, rejea swali la 4 sehemu ya kwanza) |
| 8. Unaweza kutushirikisha jinsi utaratibu wako wa huduma ya afya ya VVU ilibadilika ulipopata mtoto? |
| **KUDUMISHA HUDUMA YA AFYA** |
| 9. Sasa ningependa kujifunza juu ya matunzo yako ya afya juu ya VVU. |
| **KUFUNGA MAHOJIANO** |
| 10. Nini kingine sijakuuliza ambacho unafikiri ninatakiwa kujua? |
| Ningependa kukushukuru kwa kunishirikisha uzoefu wako leo. Je, kuna kitu kingine chochote kabla hatujafunga mahojiano yetu? |

|  |
| --- |
| **KUNDI B SWAHILI, SEHEMU YA KWANZA** |
| **SEHEMU YA KWANZA** |
| **KUJENGA MAELEWANO/ KUPELELEZA UTAMBUZI BINAFSI** |
| 1. Niambie kidogo kuhusu wewe mwenyewe. |
| 2. Niambie kuhusu shule. |
|  |
| 3. Niambie kuhusu shughuli yako ya kuingiza kipato au malengo yako ya kuingiza kipato kama bado hujaanza kufanya hivyo. |
| **HALI YA MAISHA NA HALI YA KIUCHUMI KIJAMII** |
| 4. Sasa, ningependa kusikia kuhusu hali yako ya maisha. |
|  |
| **KLABU YA VIJANA WADOGO** |
| 5. Sasa ningependa kuongelea Klabu ya vijana wadogo. |
| **KUHAMIA (PMTCT) – KUZUIA UAMBUKIZO TOKA KWA MAMA KWENDA KWA MTOTO** |
| 6. Uhamaji wa huduma ya afya ni maneno yanayotumika kuelezea pale wagonjwa vijana kama wewe wanapohama huduma toka Klabu ya Vijana kwenda kliniki ya Watu wazima. Niambie kuhusu mchakato wako wa kuhama toka Klabu ya Vijana kwenda PMTCT |
| **KUHAMIA CTC YA WATU WAZIMA TOKA PMTCT** |
| 7. Unafikiri nini kuhusu kuhama kutoka PMTCT kwenda CTC ya Watu wazima? |
| UCHUNGUZI: |
| **KLINIKI YA WATU WAZIMA KWA WATU AMBAO HAWAJAHAMIA CTC YA WATU WAZIMA** |
| 8. Ungependelea CTC yako iweje? |
| **AFYA** |
| 9. Sasa, nitataka kujua kuhusu afya yako. |
| **KIJAMII** |
| 10. Sasa ningetaka kujua kuhusu watu muhimu katika maisha yako. |
| **KUKUA** |
| 11. Sasa ningetaka kujua zaidi kuhusu kuwa kwako mtu mzima |
| **KUFUNGA MAHOJIANO** |
| 12. Ni kitu gani kingine ambacho sijakuuliza unachofikiri ninahitaji kukijua? |
| Ninapenda kukushukuru kwa kunishirikisha uzoefu wako leo. Kuna kitu kingine chochote kabla hatujafunga haya mahojiano? |
|  |
| **KUNDI B SWAHILI, SEHEMU YA PILI** |
| **SEHEMU YA PILI** |
| **KUHAMIA PMTCT** |
| 1. Mara ya mwisho, tuliongea kuhusu ni nini kilitokea kipindi cha uhamaji wako kwenda PMTCT, nani alihusika, nini kilikusisimua au kukuogopesha. Leo tungependa kujua kuhusu maoni yako juu ya mchakato wako wa kuhama. |
| - Nini mapendekezo yako kwa uhamaji kwenda PMTCT siku za baadaye kwa vijana wanaokuwa na ujauzito? |
| **MABADILIKO/MPITO** |
| 2. Mara ya mwisho, pia tuliongea kuhusu uhamaji kwenda Kliniki ya watu Wazima, ni nini umejifunza kuhusu kliniki hii hadi sasa, na ni nini kinakusisimua au kukuogopesha. Leo tungependa kujua kuhusu maoni yako juu ya mchakato wa kuhamia kliniki ya watu wazima. |
|  |
| **KLINIKI YA WATU WAZIMA** |
| 3. Unakumbuka ile CTC bora ya Watu wazima ambayo ulituelezea kwenye mahojiano mara ya mwisho? Leo tungependa kujua jinsi CTC halisi ya Watu wazima inavyofanana na hiyo klniki yako ya mfano bora. |
| **KUKUA** |
| Unakumbuka wakati tulipoongea mara ya mwisho kuhusu kuwa mtu mzima? Sasa ningependa kujua zaidi kidogo kuhusu kwamba kuwa mtu mzima kuna maana gani kwako. |
| 4. Hebu tuongee kuhusu wajibu. |
| 5. Sasa ningependa kuongelea kujitawala / uhuru binafsi. |
| **UJAUZITO** |
| 6. Unaweza kutuambia jinsi utaratibu wako wa huduma ya VVU ulivyobadilika ulipopata ujauzito? |
| **KUDUMISHA HUDUMA YA AFYA** |
| 7. Sasa ningependa kujifunza juu ya matunzo yako ya afya juu ya VVU. |
| **KUFUNGA MAHOJIANO** |
| 8. Nini kingine sijakuuliza ambacho unafikiri ninatakiwa kujua? |
| Ningependa kukushukuru kwa kunishirikisha uzoefu wako leo. Je, kuna kitu kingine chochote kabla hatujafunga mahojiano yetu |
| **KUNDI C SWAHILI, SEHEMU YA KWANZA** |
| **SEHEMU YA KWANZA** |
| **KUJENGA MAELEWANO/ KUPELELEZA UTAMBUZI BINAFSI** |
| 1. Niambie kidogo kuhusu wewe mwenyewe. |
| 2. Niambie kuhusu shule. |
| 3. Niambie kuhusu shughuli yako ya kuingiza kipato au malengo yako ya kuingiza kipato kama bado hujaanza kufanya hivyo. |
| **HALI YA MAISHA NA HALI YA KIUCHUMI KIJAMII** |
| 4. Sasa, ningependa kusikia kuhusu hali yako ya maisha. |
| **KLABU YA VIJANA WADOGO** |
| 5. Sasa ningependa kuongelea Klabu ya vijana wadogo. |
| **MABADILIKO/MPITO KWA WATU AMBAO HAWAJAHAMIA CTC YA WATU WAZIMA** |
| 6. Huduma ya afya ya mpito/mabadiliko ni maneno yanayotumika kuelezea vijana kama wewe ambao wanatoka kwenye huduma ya klabu ya vijana kwenda Ctc ya watu wazima au kliniki ya watu wazima. Unafikiri nini kuhusu kuhama kutoka PMTCT kwenda CTC ya Watu wazima? |
| **KLINIKI YA WATU WAZIMA KWA WATU AMBAO HAWAJAHAMIA CTC YA WATU WAZIMA** |
| 7. Ungependelea CTC yako iweje? |
| **AFYA** |
| 8. Sasa, nitataka kujua kuhusu afya yako. |
| **KIJAMII** |
| 9. Sasa ningetaka kujua kuhusu watu muhimu katika maisha yako. |
| **KUKUA** |
| 10. Sasa ningetaka kujua zaidi kuhusu kuwa kwako mtu mzima |
| **KUFUNGA MAHOJIANO** |
| 11. Ni kitu gani kingine ambacho sijakuuliza unachofikiri ninahitaji kukijua? |
| Ninapenda kukushukuru kwa kunishirikisha uzoefu wako leo. Kuna kitu kingine chochote kabla hatujafunga haya mahojiano? |
|  |
| **KUNDI C SWAHILI, SEHEMU YA PILI** |
| **SEHEMU YA PILI** |
| **MABADILIKO/MPITO** |
| 1. Mara ya mwisho, pia tuliongea kuhusu uhamaji kwenda Kliniki ya watu Wazima, ni nini umejifunza kuhusu kliniki hii hadi sasa, na ni nini kinakusisimua au kukuogopesha. Leo tungependa kujua kuhusu maoni yako juu ya mchakato wa kuhamia kliniki ya watu wazima. |
| **KLINIKI YA WATU WAZIMA** |
| 2. Unakumbuka ile CTC bora ya Watu wazima ambayo ulituelezea kwenye mahojiano mara ya mwisho? Leo tungependa kujua jinsi CTC halisi ya Watu wazima inavyofanana na hiyo klniki yako ya mfano bora. |
| **KUKUA** |
| Unakumbuka wakati tulipoongea mara ya mwisho kuhusu kuwa mtu mzima? Sasa ningependa kujua zaidi kidogo kuhusu kwamba kuwa mtu mzima kuna maana gani kwako. |
| 3. Hebu tuongee kuhusu wajibu. |
| 4. Sasa ningependa kuongelea kujitawala / uhuru binafsi. |
| UCHUNGUZI: |
| **MALEZI** (kama hana watoto, ruka kipengele hiki cha MALEZI, rejea swali la 4 sehemu ya kwanza) |
| 5. Unaweza kutushirikisha jinsi utaratibu wako wa huduma ya afya ya VVU ilibadilika ulipopata mtoto? |
| **KUDUMISHA HUDUMA YA AFYA** |
| 6. Sasa ningependa kujifunza juu ya matunzo yako ya afya juu ya VVU. |
| **KUFUNGA MAHOJIANO** |
| 7. Nini kingine sijakuuliza ambacho unafikiri ninatakiwa kujua? |
